# Supplementary material for: A New Inert Natural Deep Eutectic Solvent (NADES) as a Reaction Medium for Food-Grade Maillard-Type Model Reactions
Source: Foods. 2023 May 2;12(9):1877. doi: 10.3390/foods12091877 (PMC10178046; doi:10.3390/foods12091877)
Supplement: Supplementary file 1 [file foods-12-01877-s001.zip › foods-2313051-supplementary.pdf]

Supplementary Data

Table S1: UHPLC-MS/MS validation results consist of five recovery rates from 750 – 30000 nmol/L, intraday precision, the limit of detection (LoD), and limit of quantification (LoQ) in a solvent mixture of (80/20; v/v; water/methanol) for sucrose/D-sorbitol [nmol/L].

	**sucrose**	**D-sorbitol**
Recovery 1 (750 nmol/L)	118.8 ± 15.5%	80.6 ± 8.9%
Recovery 2 (2000 nmol/L)	111.2 ± 3.2%	83.2 ± 2.5%
Recovery 3 (6000 nmol/L)	113.6 ± 3.0%	92.0 ± 2.8%
Recovery 4 (10000 nmol/L)	102.2 ± 2.6%	95.2 ± 2.0%
Recovery 5 (30000 nmol/L)	81.8 ± 4.1%	105.3 ± 2.8%
Intraday precision	5.7%	3.8%
Limit of detection (LoD)	< 3.05 nmol/L	17.92 nmol/L
Limit of quantification (LoQ)	< 9.15 nmol/L	53.76 nmol/L

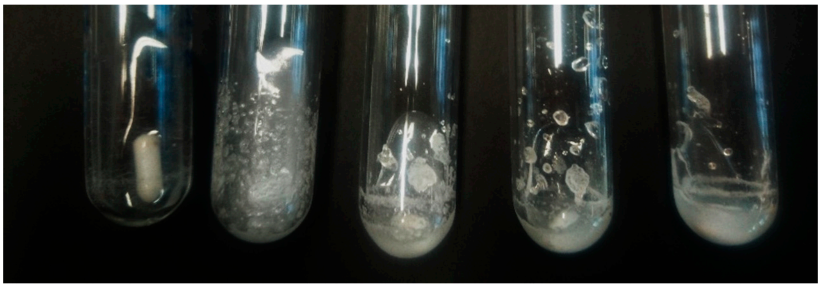

(1:1:8) (1:1:7) (1:1:6) (1:1:5) (1:1:4)

Figure S1: Selected Suc/Sorb NADES system produced by the heating method with different water contents (molar ratio of sucrose: D-sorbitol: water).

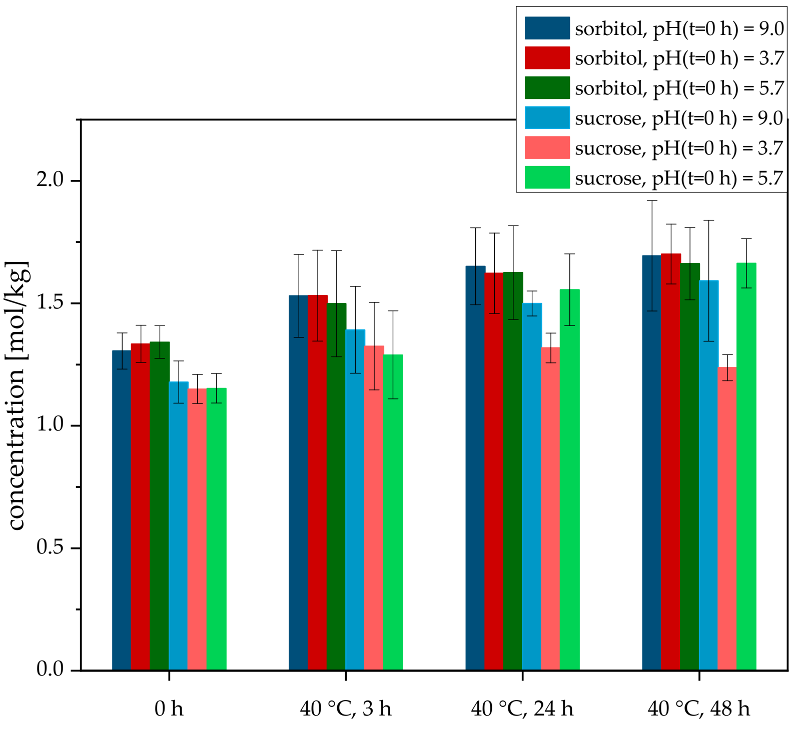

Figure S2: pH stability of Suc/Sorb NADES system by quantification of sucrose and D-sorbitol before and after heat treatment at 40 °C (0 h, 3 h, 24 h, 48 h) at different pH values (t= 0 h: 3.7 (red); 5.7 (green), 9.0 (blue); t= 48 h: 3.5 (red), 6.0 (green), 8.5 (blue)). Control is for both NADES ingredients calculated as 1.5 mol/kg.

Table S2: Concentration of *Maillard* model reaction products produced in different solvents (**(*R*)-2**, **(*S*)-2** in Suc/Sorb NADES, Suc/Glc NADES, Betaine/GlyOH NADES) referred to mmol of the educt D,L-glyeraldehyde and **1** (in Suc/Sorb NADES and 0.03 mmol/L phosphate buffer in µmol/g) as the mean of three reproduced replicates and three technical replicates with corresponding standard deviation.

**solvent**	***S*-2 [µmol/mmol]**	***R*-2 [µmol/mmol]**	**Sum [µmol/mmol]**
Suc/Sorb NADES	90.35±7.59	71.36±4.21	161.72±11.80
Suc/Glc NADES	91.00±6.16	75.97±5.62	166.97±11.78
Betaine/GlyOH NADES	48.42±3.97	32.21±3.29	80.63±7.26
	**1** [µmol/g]		
Suc/Sorb NADES	95.70±12.66		
0.03 mmol/L phosphate buffer	10.42±1.13		
